# Teaching students to R^3^eason, not merely to solve problem sets: The role of philosophy and visual data communication in accessible data science education

**DOI:** 10.1371/journal.pcbi.1011160

**Published:** 2023-06-08

**Authors:** Ilinca I. Ciubotariu, Gundula Bosch

**Affiliations:** 1 Department of Biological Sciences, Purdue University, West Lafayette, Indiana, United States of America; 2 Department of Molecular Microbiology and Immunology, Johns Hopkins Bloomberg School of Public Health, R^3^ Center for Innovation in Science Education, Baltimore, Maryland, United States of America; SIB Swiss Institute of Bioinformatics, SWITZERLAND

## Abstract

Much guidance on statistical training in STEM fields has been focused largely on the undergraduate cohort, with graduate education often being absent from the equation. Training in quantitative methods and reasoning is critical for graduate students in biomedical and science programs to foster reproducible and responsible research practices. We argue that graduate student education should more center around fundamental reasoning and integration skills rather than mainly on listing 1 statistical test method after the other without conveying the bigger context picture or critical argumentation skills that will enable student to improve research integrity through rigorous practice. Herein, we describe the approach we take in a quantitative reasoning course in the R3 program at the Johns Hopkins Bloomberg School of Public Health, with an error-focused lens, based on visualization and communication competencies. Specifically, we take this perspective stemming from the discussed causes of irreproducibility and apply it specifically to the many aspects of good statistical practice in science, ranging from experimental design to data collection and analysis, and conclusions drawn from the data. We also provide tips and guidelines for the implementation and adaptation of our course material to various graduate biomedical and STEM science programs.

This is a *PLOS Computational Biology* Methods paper.

## Introduction

In the past decades, there has been a growing acknowledgement of the need to improve the practice of science through better education in research integrity: Responsibility, Rigor, and Reproducibility—the 3 “R” core norms of good science [1–5]. For instance, at the Johns Hopkins Bloomberg School of Public Health, in the R^3^ Center for Innovation in Science Education (R^3^ISE), we produce graduate programs and curricula focusing on those core principles by including formal training in critical thinking across research disciplines, ethics in science and society, the nature and sources of mistakes, and responsible science communication [5–7]. Among the tenets of good research conduct fall good statistical practice, which encompasses the full data pipeline from experimental design and conduct to data collection and curation, data analyses, interpretation of results in context, full reporting, and appropriate quantitative and logical understanding [8,9].

Earlier previously published work has emphasized the separation between statistics and biology course work at the undergraduate level [10], and biology students lacking statistical training [11] can lead to statistical errors made by biologists at the professional stage, including statistical errors in high-impact journals [12,13]. There were many calls to change statistical training broadly with approaches like including problem solving [14], incorporating statistics into biology curricula [12,15], and developing data science initiatives for life science education [16]; however, this effort was concentrated at the college level.

At the graduate level, in our experiences [5] and those of others [17,18], there exist learning gaps in statistical training in the sciences, and this is often the first time these students receive advanced training in statistics [19]. For instance, in numerous seminars, committee meetings, and oral exams that we and colleagues attended (Personal Communications, Gundula Bosch), we frequently observed students uncritically operating statistical software, proving incapable of providing sound rationales for the application of a particular method or just making sense of statistical data. Thus, our motivation is to train students who needed to learn the basics of data science in their field to properly reason, not merely to solve problem sets. This has led us to develop a course for biomedical graduate learners, where we adapted our pedagogy approach in quantitative reasoning to include philosophical elements in statistics through exercises in reasoning and error reduction in statistical logic. Theoretically, this course could also be of particular utility to advanced-level undergraduate students interested in STEM careers, although an in-depth knowledge of public health and biomedical sciences is highly encouraged because many of the manuscripts we use as examples and resources in the course require a bit of background knowledge and topic understanding. Nonetheless, the foundational course material could be adapted and taught for other cohorts of interest.

This commentary provides information about our general approach and tips for other educators on how to adapt it to develop their own course modules in a similar fashion (Table 1). Our overall goal is to help improve research integrity through teaching good research practices, while including material centered around errors and fallacies that teach not only technical skills but also aspects of applied logic and ethics training, as well as fundamentals of responsible communication and data visualization.

**Table 1:** Foundational course materials and general tips for course modules.

Session title	Lecture content overview and error concepts addressed	Topics for activities and discussions
**Responsible communication of statistical information along data science life cycle**	• Importance of truthful and effective ways to communicating data science information in biomedicine. • Impact of misleading data science communication. • Ethical implications of effective data communication illustrated through examples from John Snow’s historic cholera map [33].	• Provide an example of what you consider either effective or poor data communication from your own field of work, thereby using articles, the media, etc. to illustrate your argument (post the graph from the source into your response). Explain what the graph displays or what it should convey. • How would you communicate the main message from the graph to a non-scientist?
**The philosophy behind sound hypothesis testing—more than just a statistical method**	*p*-values: • The philosophical underpinnings of testable hypotheses and testing methods. Use and misuse of *p*-values in the biomedical data sciences. Importance of other metrics in this context, e.g., statistical power in relation to sample size, effect sizes, and their role in the *p*-value debate.	• Individual reflection: apply the principles from Fisher’s work [44] to your research and design an experiment—formulate the appropriate elements of hypothesis testing and describe the experimental approach you would use to answer the proposed question. • Individual or group activity: find an article from your field of interest and critique the responsible use or misuse of the *p*-values, as well as the considerations of statistical power and sample size in the article.
**Causal inference research and the role of chi-square tests**	• Logical misconceptions in biomedical reasoning—evaluating statistical evidence in causation research.	• Individual problem sets on spurious (but not obvious) correlations [85]: applying the chi-square method to test whether a claim for causation is justifiable. • Group discussion evaluating a claim for causality in an exercise derived from real-word research: applying chi-square statistics to HBV infection data to check if sex and/or age play a causal or correlational role in HBV infection [86]. • Extra discussion: What implications could the statistical fallacy of false causality have, especially in the context of clinical research?
**Investigating research (ir)reproducibility**	• Explanation of ANOVA testing [87], pitfalls of variance testing [88], variance/ANOVA in the context of reproducibility [39], and applications of variance tests to replicability studies.	• Using examples from “omics” research or population-based vaccination studies [89–91], students learn to describe characteristics of well-defined statistical designs and parametric assumptions that data must satisfy for ANOVA variance tests before proceeding to do calculations. • Additional group discussions circle around identification of poorly selected nonparametric assumptions as well as potential pitfalls of ANOVA variance testing that could be harmful to research and reproducibility?
**Big data interpretation—improving how data are visualized and what “data stories” can be accurately told through graphical representations**	Errors and fallacies in reasoning using large data sets.	Individual (final) project work: Students: • Sign up for a provided data visualization example (graph) and critique it with respect to legibility, ability to convey a message effectively and clearly, and potential logical and statistical fallacies. • Improve the graph and provide a reasoning for your new visualization design and method. • Record a maximum 3-min “Ted Talk” style talk and present a “before and after” graph example from the above exercise for displaying a big data set. • Explain the context of the graph, thereby justifying their choice of graphical design update.

### Curriculum overview

#### Data science education from errors points of view

The lack of reproducibility of key scientific findings has been a growing concern across many disciplines, in tandem with retractions [20,21]. The reasons behind the reproducibility debate are multifaceted, ranging from insufficient training in rigorous research methods, to sloppy literature outputs, to outright misconduct [22–25]. Additionally, logical fallacies, statistical mistakes during data analysis and interpretation, inadequate reporting of statistical techniques, and erroneous communication are other important contributing factors. For instance, in fields like neuroscience, cell biology, and medicine (but certainly not limited to these disciplines), numerous papers were found to have used inappropriate statistical techniques to compare experimental effects [26], had invalid statistical analyses [27], or contained multiple statistical flaws [28,29].

Hence, the rationale to approach data science education from an error point of view stems from this literature-supported experience that mathematical and statistical mistakes make up for a significant portion of mishaps in scientific practice [30]. In the R3 program across all of our courses, we refer to “errors” in science broadly as mistakes that stem from conceptual misunderstandings, lack of good practice skills in hypothesis testing, data analysis and management, as well as miscommunication [1]. This approach is broader than the statistical understanding of Type 1 or Type 2 errors, and although it allows us to cover those 2 specific errors in our quantitative reasoning course, we also cover other errors and mistakes in science that may occur in the statistical realm. For instance, through the literature, it is clear that there are a multitude of errors that could occur throughout the entire statistical process from the design of a study to data analysis, to the documentation of the application of statistical methods, to the presentation of study data, and finally to the interpretation of study findings. In other words, this could include a range of errors from failure to use and report randomization, to not providing test assumptions, to using presenting *p*-values as results and in interpretation [29].

Incorporating this lens in our course allows us to focus on science correction and create discussion for scientific advancement [31].

#### Teaching approach

The format of the course is balanced between short-style lectures, fireside chats with experts in the field to bring some of these statistical concepts to life, and various discussions and activities (see Table 1 for more details). The activities are applications of the lectures in some form, but also have a few specific learning objectives, depending on the topic discussed at hand in a respective week. The students discuss the use of statistical tests and can understand the tests, although we don’t place great emphasis on actual calculations. They also become versed in reading the scientific literature with examples of errors and statistical tests in practice and application. Further, we encourage the use of software like R, Tableau, or Excel for preparing the data visualization exercises (since this program is housed in the School of Public Health, we usually have students across departments and at different levels of expertise with programming languages and skills, so we provide some examples through the course material). Below, we describe our material in detail and present the activities and discussions we incorporate in our lessons.

#### Hypothesis testing and *p*-values

First, we begin with the integration of data science into the scientific life cycle [32] and connect this to effective data visualization and storytelling through graphical representations. We travel through time from John Snow’s maps of the 1854 Broad Street Pump cholera outbreak [33] to the present day with examples of SARS-CoV-2 graphical summaries of research that were not the most effective in conveying important data and in turn, public health action [34–37]. We then introduce a hypothesis testing framework, which serves as a guide for all steps in research, from establishing a research question, identifying appropriate statistical tests, interpreting a test statistic outcome, and communicating one’s findings.

Hypothesis testing conceived by Neyman and Pearson, in which a researcher considers null and alternative hypotheses to answer a scientific question, can lead to errors like Type 1 and Type 2, when a null hypothesis is rejected when true or when a null hypothesis is not rejected when false, respectively [38]. In our discussion of hypothesis testing, we emphasize the importance of planning an experiment and teach practice applications of common tests like *t* tests, ANOVA, and post hoc tests, thereby focusing on the reasoning aspect behind the applications. We also provide examples from the literature with respect to potential errors that might occur in the context of hypothesis testing (such as using unpaired tests for paired data, using unequal sample sizes for paired *t* tests), thereby emphasizing the “error lens.”

Moreover, we highlight the need for clear and sufficient description in publication [39], as this information is key for the transparent reporting element of responsible science communication.

Interwoven into the theory of hypothesis testing from a problem-centric point of view is the *p*-value debate that provides a measure of the strength of evidence against a null hypothesis and which has stirred much controversy due to its improper use [40–42].

“*The p-value was never intended to be a substitute for scientific reasoning*.*”–Ron Wasserstein* [43]

We take a philosophical approach when teaching *p*-values, starting from the depths of Fisher’s initial work in which he described their use as an index for measuring the strength of evidence against the null hypothesis [44]. Using an error lens, literature examples help us illustrate instances of less rigorous or improper research practice, and misuse of *p*-values and statistical inference [8,45–47]. We describe the recent state of the field as presented by statisticians through various pieces of scientific literature [8,46,48–52], with the argument that general misuse of statistical methods and *p*-values have played a large part in science’s reproducibility crisis [8,47,53]. Further, we discuss the various perspectives [54] centered around the *p*-value debate, including the opinion that *p*-values may not be a useful metric even when used “correctly,” the view that *p*-values should be used in tandem with other metrics such as confidence intervals, etc., the suggestion that the “gold-standard” of the *p*-value threshold should be redefined to 0.005 [8,50,55–57]. The *p*-value has been reinterpreted as an “observed error rate” and as measure of evidence; it is not used today as it was initially intended [42,58,59] and it shouldn’t represent the be-all and end-all value of studies—although in many cases, it does.

“*Over time it appears the p-value has become a gatekeeper for whether work is publishable*, *at least in some fields*.*”–Jessica Utts* [43]

#### Null results, publication bias, HARKing, p-hacking, and more

Related to hypothesis testing, we highlight the concept of “null results” or experiments that produce results that do not support a hypothesis made prior to conducting the study [60]. Many times, null findings do not end up published in the scientific literature, which represents publication bias or the trend of journals to prioritize positive findings for publication—one of the 4 horsemen of the reproducibility apocalypse [61–63].

For instance, a study of social science experiments found that majority of null results, in contrast to “strong” results which are usually published in top journals, remain unwritten [60,64]. Also called the “file drawer phenomenon” [65], this can be detrimental to the scientific community, as null results may not be empty of biological meaning and can be as informative as “statistically significant” results that lack replication [66].

This also accentuates the “pressure to publish” that affects the quality of rigorous research and can lead to instances of irreproducibility with practices like *p*-hacking, selective reporting, or data dredging [67]. This refers to performing statistical analyses continuously until nonsignificant results become significant and reporting only significant results [68,69]. Similarly, HARKing, or hypothesizing after results are known [70], is another questionable research practice that we emphasize in our teaching through fireside chats and case studies we have developed with short examples related to biomedical sciences. We also include other issues such as the multiple testing problem or considering a set of statistical inferences simultaneously.

In our course, we provide existing proposals and available solutions to addressing these issues such as the recommendation of “results blind evaluation” of manuscripts [71]. Recently, some journals are moving in the direction of welcoming the publication of negative findings and recognize their value in addressing important questions while being methodologically robust [61]. In another effort to combat this problem, researchers can preregister their studies or write registered reports [72], which allows for preconditioned acceptance after an initial peer review prior to data collection, thus emphasizing study design and sound methodologies rather than “significant” results. Not surprisingly, this has so far been shown to reduce publication bias and increase replication studies [73,74].

We teach that *p*-values cannot be interpreted in isolation, and statisticians have offered suggestions in this direction for many years; for instance, instead of thresholding, context is needed for interpretation with other robust measures such as sample size, effect sizes, and confidence intervals [51,52,75–77].

Another example of a session in which we relate content material to potential consequences of error or misuse is a discussion of chi-square test for independence, Pearson correlations, and the logical fallacy of false causality. *Cum hoc ergo propter hoc* indicates that tests which are meant to present relationships between variables or correlations should not be used to establish a cause-and-effect relationship, and we ask students to provide examples from the scientific literature.

The overarching debate over the *p*-value and other elements clearly shows that institutional changes are needed, including better training and educational resources that highlight reasoning and error reduction, and this is what our course aims to accomplish [9,53,78].

### Responsible communication—Essential to scientific reasoning

As part of the R3 program, we have previously highlighted the importance of rigorous research and presented our framework for responsible scientific communication training as means by which research integrity can be improved [7]. Responsible science communication, which includes value-based principles built on established ethical principles, such as honesty, stewardship, objectivity, openness, accountability, and fairness, encompasses processes from the lab bench to the dissemination of scientific work [7]. By including the philosophical elements of reasoning and error reduction in statistical training, we also realized that now more than ever, visualization is a form of responsible scientific communication; hence, we included it as a connecting thread through our lectures.

Throughout the SARS-CoV-2 pandemic, it has become much clearer that responsible scientific communication is necessary to translate research into public health practice and this includes accurate representation and display of data. Data display is a form of visual communication, and this tool is useful when reaching out to the public and conveying research findings to bring data to life. There have unfortunately been many examples of poor graphical representation of data, ranging from inappropriate graph choice to wrongly scaled axes, and to nondigestible information. We reflect on these in the course and ask students to reconstruct data display in new form through guidelines we cover in lectures.

To this end, one important exercise we highlight throughout the course (see Table 1) is the ability to responsibly communicate data to both one’s research colleagues with STEM-focused training and non-scientists. We ask our students to pick specific examples of both effective and ineffective presentation of data from the literature and briefly explain the main message of the selected plots. This exercise, while challenging, is rewarding because it allows the students to reflect on how to improve both their data representation and their communication—parts that are both essential for furthering science and its results.

The themes of responsible science communication and data visualization represent 2 examples of continuity elements which we revisit in multiple course sessions, and we believe highlighting an exercise throughout the course in various forms and contexts prepares the students to profoundly grasp a concept such as hypothesis testing. Another example we review is that of *p*-values, through older studies that have misused this metric and both the current biomedical literature (see Table 1 for details).

### Course learning outcomes: Results and reflections

Prior to the implementation of this reformed quantitative reasoning course with an error-focused lens, and with particular emphasis on visualization and communication competencies, we offered a course that was much more focused on rote statistical tests and concepts. As we collected students’ impressions of the 2 courses offered a year apart, we have a comparative “before and after” assessment and can summarize findings from a combination of student evaluation and surveys from the course prior to its new approach and after implementation with the new lens. We can thus provide a better sense of the outcome of students’ experiences following the implementation of this didactic approach to a cohort of graduate students in the R3ISE program. Many students claimed that the previous course did not improve their confidence, while based on the course evaluation, the students appreciated this new lens on data visualization, communication, and interpretation, claiming that the course “looked at stats in a different way” and “helped [us] critically assess data [while] providing foundational knowledge of statistical analysis and data visualization”. Further, when asked whether the students of this new pedagogical approach felt that they achieved the course objectives as designated in the syllabus, there was improvement (from a mean of 3.3 to 3.9, on a scale of 1 to 4, with 1 representing “pool” and 4 representing “excellent”), and similarly when asked if they gained more expertise in the targeted skills of reasoning and data communication (3.1 increased to 3.9). Additionally, based on students’ performances on the various assignments described in Table 1, it was clear that this new approach made the material more engaging and digestible for students, who performed better and were able to answer the questions correctly 90% of the time as opposed to 74%. For this new course, the students indicated that examples were “practical” and “reproducible,” which strengthens our argument for good scientific practices and application. Overall, students claimed that the course was “useful” and this was the first time the concepts covered were “used and interpreted in a meaningful way.” Further, multiple students “appreciated the depth” of the course, especially the “experience with visualization and analyzing biology-related data.” Based on these observations, the strong focus on philosophy and visualization/communication was effective in achieving the main targets of the course and lastly, we copy some direct points from the student survey on achieved objectives:

○ *I gained a better outlook on statistical significance and biological relevance*.
○ *It greatly improved my quantitative reasoning skills*.
○ *[I am] definitely more appreciative of statistics and reasoning now*. *This course allowed me to understand and truly reflect on the “why” behind each statistical test/term*.
○ *[The course] made me think more critically about the tests I can perform on data*.
○ *[The course] Made me more wary of data manipulation to fit a hypothesis*.
○ *[I] Understand the p-value debate and how context is ultimately important for study results in biomedical science*.
○ *[The course] Made me more aware of the different statistical tools available to me and their potential uses and shortcomings*.
○ *Now [I] am more confident in my quantitative reasoning abilities and have skillset to use in my upcoming research*.

Surely, we recognize that our program (including this revised course) is still in its young stages, although based in design and structure on established educational theory, and therefore more data collection on program effectiveness is ongoing. We aim to continue collecting data and further understand the impact of such a course on students’ performances in acquiring the target competencies and even plan to compare with other graduate training programs based on measurable outcomes.

Furthermore, in addition to making this data publicly available, our goal is also to make our course materials and resources (such as modules, lesson plans, and fireside chats) available for other institutions to adapt to their biomedical science training programs (see our corresponding GitHub repository: https://github.com/JHU-R3ISE).

## Conclusions

More work is needed to enhance quantitative and statistical training education for graduate students in the science disciplines [79]. Here, we described and propose our approach to tackling this issue of deficit in quantitative reasoning training by means of a course with an error-focused lens that can be molded and adapted to particular student cohort needs.

*When training scientists in the use of quantitative methods*, *I and others often feel pressure to teach the standard approaches that peers and journals expect rather than to expose the problems*.*–Steven Goodman* [80]

In a sense, we have implemented the familiarly known Bloom’s Taxonomy [81,82] for teaching, encompassing major categories of comprehension through our lectures of error concepts, application of learned material to students’ specific research contexts, analysis through abstracted case examples, evaluation by means of critiquing provided cases, and creation through a final project bringing together all elements in a Ted-style talk. We provide tips for planning and implementation for other educators so such a course can be integrated into graduate science curricula.

Real-world data sets and cases of problems can be easily extracted from resources like Retraction Watch (retractionwatch.com) or other publicly available databases as the basis for case scenarios with problems and discussion questions (Table 1).

We recognize that other trainers in statistics may have described similar approaches in the past, especially with regards to hypothesis testing and *p*-values; for instance, books such as *Common Errors in Statistics (and How to Avoid Them)* [83] and *Statistics Done Wrong*: *The Woefully Complete Guide* [84] address popular mistakes in data collection, discuss statistical blunders in modern science, and provide information on appropriate statistical technique, and are thus excellent resources for our class. To our knowledge, there has not been any published comprehensive course approach, and guide format such as this one, and so we aim for such a course to prevent gaps in quantitative reasoning and enable our students to feel prepared in their understanding of statistical test applications and visualization and communication, which we argue are key elements of responsible science communication. The specific focuses on the philosophical bases of elements like hypothesis testing, in combination with data visualization and responsible communication which we implement in our course, is what sets this work apart from the traditional approach to teaching data science material. We believe this work could be taken as a foundational approach and adapted to specific departments for teaching data science across STEM-focused disciplines to lab scientists, public health practitioners, etc.
